# Clinical Features of a Retinopathy Associated With a Dominant Allele of the *RGR* Gene

**DOI:** 10.1167/iovs.18-25061

**Published:** 2018-10

**Authors:** Rola Ba-Abbad, Monique Leys, Xinjing Wang, Christina Chakarova, Naushin Waseem, Keren J. Carss, F. Lucy Raymond, Kinga M. Bujakowska, Eric A. Pierce, Omar A. Mahroo, Moin D. Mohamed, Graham E. Holder, Marybeth Hummel, Gavin Arno, Andrew R. Webster

**Affiliations:** 1 UCL Institute of Ophthalmology, London, United Kingdom; 2 Moorfields Eye Hospital, London, United Kingdom; 3 WVU Eye Institute, West Virginia University, Morgantown, West Virginia, United States; 4 Genetics Laboratory, Department of Pediatrics, University of Oklahoma Health Sciences Center, Oklahoma City, Oklahoma, United States; 5 Department of Haematology, University of Cambridge, Cambridge, United Kingdom; 6 NIHR BioResource-Rare Diseases, University of Cambridge, Cambridge, United Kingdom; 7 Department of Medical Genetics, University of Cambridge, Cambridge, United Kingdom; 8 Massachusetts Eye and Ear Infirmary, Ocular Genomics Institute, Boston, Massachusetts, United States; 9 Harvard Medical School, Boston, Massachusetts, United States; 10 Department of Ophthalmology, St. Thomas' Hospital, London, United Kingdom; 11 Department of Ophthalmology, National University of Singapore, Singapore; 12 Department of Pediatrics, Section of Medical Genetics, West Virginia University, Morgantown, West Virginia, United States

**Keywords:** autosomal dominant retinal dystrophy, deep retinal reticular pigmentation, next generation sequencing, whole genome sequencing, *RGR*

## Abstract

**Purpose:**

We describe the clinical features in two pedigrees with dominantly inherited retinopathy segregating the previously reported frameshifting mutation, c.836dupG (p.Ile280Asn*78) in the terminal exon of the *RGR* gene, and compare their haplotypes to that of the previously reported pedigree.

**Methods:**

The probands were ascertained at West Virginia University Eye Institute (WVU) and Moorfields Eye Hospital (MEH) through next generation sequencing (NGS) and whole genome sequencing (WGS) respectively. Clinical data included visual acuity (VA), visual fields, fundus autofluorescence (FAF), optical coherence tomography (OCT), and electroretinography (ERG). Haplotype analysis was performed using Sanger sequencing of the DNA from the molecularly ascertained individuals from the three pedigrees.

**Results:**

Nine heterozygous mutation carriers were identified in two families. Four carriers were asymptomatic; five carriers had variable VA reduction, visual field constriction, and experienced difficulty under dim illumination. Fundus examination of the asymptomatic carriers showed diffuse or reticular pigmentation of the retina; the symptomatic carriers had chorioretinal atrophy. FAF imaging showed widespread signal loss in advanced retinopathy, and reticular hyperautofluorescence in mild cases. OCT showed loss of outer retinal lamina in advanced disease. ERG showed moderate-to-severe rod–cone dysfunction in two symptomatic carriers; and was normal in three asymptomatic carriers. A shared haplotype flanking the mutation of up to 6.67 Mb was identified in both families. Within this region, 1.27 Mb were shared with the first family reported with this retinopathy.

**Conclusions:**

The clinical data suggest a variable and slow degeneration of the RPE. A shared chromosomal segment surrounding the *RGR* gene suggests a single ancestral mutational event underlying all three families.

Inherited retinal dystrophies are a group of disorders in which retinal dysfunction and/or degeneration is due to disease-causing variants in one or both alleles of a single gene. They present with variable clinical features, and have high genetic and allelic heterogeneity. To date, disease-causing variants in more than 300 genes have been identified (available in the public domain at https://sph.uth.edu/retnet/; The University of Texas-Houston Health Science Center, Houston, TX, USA). Proteins encoded by these genes can be expressed ubiquitously, or by specific retinal cell types.^1–4^

The retinal G-protein coupled receptor gene (*RGR,* MIM_600342) is located on chromosome 10q23.1, and encodes an intracellular opsin localized to the retinal pigment epithelium (RPE) and Müller cells.^5–7^ To date, only one disease-causing mutation in *RGR* has been described: a heterozygous frameshifting mutation (chr10:86018343dupG; NM_002921.3:c.836dupG; p.Ile280Asn*78 — the same mutation as reported previously), which was reported in a pedigree with autosomal dominant retinopathy, but the clinical data were limited (RP44; MIM-613769).^8^ An allele reported to be associated with recessive retinal disease has been re-evaluated, with the disorder in those patients explained, instead, by biallelic mutation of *CDHR1*.^9^ Therefore, the consequences of biallelic loss of function of *RGR* on the human retina remain unknown.^9^ We characterized the clinical features secondary to the frameshifting mutation p.Ile280Asn*78 in *RGR* in two not knowingly related Caucasian pedigrees from the United Kingdom and United States.

## Methods

**Figure 1:**
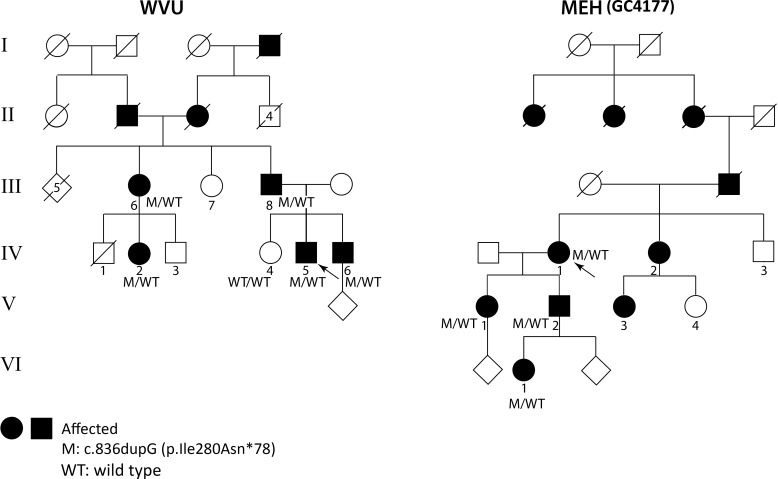
Pedigrees of the families from WVU and MEH-GC4177. Subjects with history of blindness in WVU: I, II, and MEH II, III, and IV are shown as solid symbols. In both pedigrees, all the genotyped members (denoted as M/WT for heterozygotes and WT/WT for those with wild type alleles) were examined and retinal images were obtained. Subjects WVU: IV-2, IV-5; and MEH-GC4177: V-2, VI-1 had distinct retinal changes on imaging but no visual symptoms. Arrows indicate the proband in each family.

Two probands (shown by arrows in Fig. 1) were initially ascertained through large sequencing studies. Family WVU was recruited from the medical retina clinic at the West Virginia University Eye Institute (WVU); family GC4177 was recruited from the inherited retinal disorders clinic at Moorfields Eye Hospital (MEH; Fig. 1). After obtaining informed consent, the probands and their family members donated blood for genetic testing. DNA analysis from the WVU family was performed as part of the National Ophthalmic Disease Genotyping and Phenotyping Network (eyeGENE) study, as a Health Insurance Portability and Accountability Act (HIPAA)-compliant study that was approved by the institutional review boards at WVU, and the National Eye Institute (Bethesda, MD, USA). DNA analysis from the GC4177 family was performed as part of the National Institute for Health Research BioResource - Rare Diseases (NIHRBR-RD) study, which was approved by MEH Research Management Ethics, and Cambridge South Research Committee, and adhered to the tenets of the Declaration of Helsinki. Details of the molecular investigations at WVU and MEH, including the haplotype analysis from the two pedigrees and the family reported by Morimura et al.,^8^ are provided in the Supplementary Material.

Sanger sequencing of all coding exons and exon-intron boundaries of *RGR* in 18 unrelated probands from MEH, whose retinal features were similar to those of the MEH proband and WVU: III-8, and were negative for *CHM* mutations, did not identify further carriers of this change or other likely disease-causing variants in *RGR* (primers available upon request).

The probands and available family members underwent ophthalmic examination including best-corrected Snellen visual acuity (VA), dilated fundus examination, optical coherence tomography (OCT), fundus autofluorescence (FAF) imaging (Spectralis HRA+OCT; Heidelberg Engineering GmbH, Heidelberg, Germany), color fundus photography, Goldmann perimetry, and electrophysiologic testing using protocols that incorporated the recommendations of the International Society of Electrophysiology of Vision (ISCEV) for electroretinography (ERG), pattern electroretinography (PERG), and electrooculography (EOG) at the time of examination.^10–12^

## Results

### Molecular Genetic Analysis

A previously reported^8^ heterozygous variant in *RGR* (NM_002921.3 c.836dupG; p.Ile280Asnfs*78, MIM_600342.0002) was detected in the genomic DNA from both probands WVU-IV:5, and GC4177-IV:1 by targeted next generation sequencing (NGS) and whole genome sequencing (WGS), respectively. Direct Sanger sequencing of the DNA from available family members from both pedigrees (Fig. 1; WVU: III-6, III-8, IV-2, IV-5, IV-6, and GC4177: V-1, V-2, and VI-1) demonstrated the variant to be present in the DNA from all of these individuals, and segregate with retinal changes on fundus examination; WVU: IV-4 had normal ocular examination and the wild type allele. Haplotype analysis using 32 single nucleotide variants (SNV), with a minor allele frequency (MAF) of <0.2 in the gnomAD dataset (available in the public domain at http://gnomad.broadinstitute.org; Broad Institute, Cambridge, MA, USA), selected from the WGS data of GC4177: IV-1 showed eight SNVs to be shared between the following subjects: GC4177: V-1, V-2 and WVU: IV-5, IV-6 (Table 1), indicating a shared haplotype region of 6.67 Mb inclusive of *RGR* (primers available upon request). Additional Sanger sequencing of the DNA from three affected and two unaffected family members from the first pedigree, which was reported by Morimura et al.,^8^ for the eight shared SNVs showed a common haplotype of 1.27 Mb across the three pedigrees.

**Table 1:**
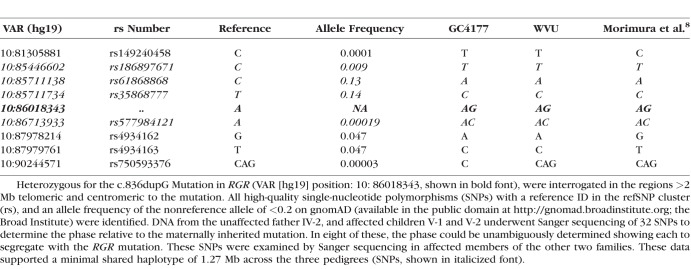
Whole Genome Sequencing Data for the Proband GC4177-IV-1

The variant, a duplication of a single nucleotide (G) at position 836 in exon 7 of *RGR,* is predicted to result in an out-of-frame extension through the canonical stop codon, producing a longer polypeptide with 77 novel amino acid residues at the C-terminal end, and replacing 16 amino acid residues in the cytoplasmic domain of RGR. Because the variant is in the terminal exon of *RGR,* the transcript is likely to evade nonsense mediated decay and a mature protein could be produced.

### Clinical Features

The clinical, visual function and imaging data are summarized in Table 2. Nine subjects were examined, five from two generations in the WVU pedigree and four from three generations in the GC4177 pedigree (Fig. 1). No other family members were available for examination. Median age of the asymptomatic carriers with normal visual acuity was 50 years (range, 23–60 years). There was no contributory medical or drug history.

**Table 2:**
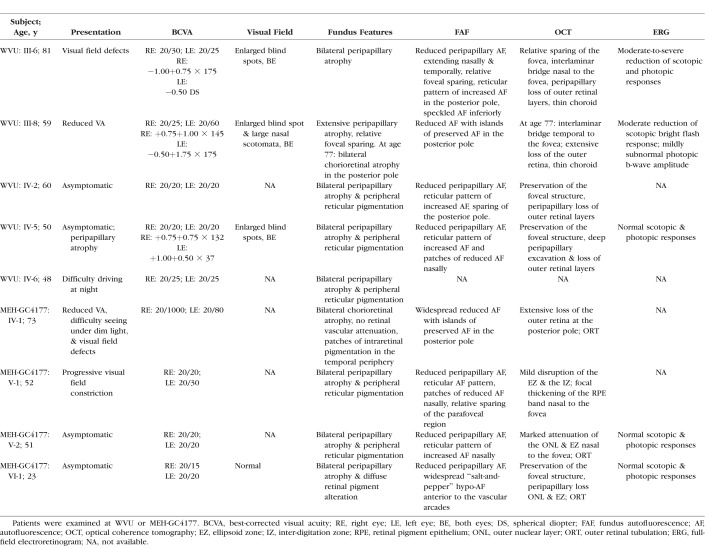
Clinical Features of RGR Retinopathy in Two Unrelated Pedigrees

The earliest symptoms in WVU: III-8 were visual field defects and reduced VA at the age of 59 years. WVU: III-6 had visual field defects and mildly reduced VA at the age of 81 years. Subjects GC4177: IV-1 and GC4177: V-1 first noticed progressive visual field constriction during their third decade of life. The VA in symptomatic carriers ranged between 20/20 and 20/1000. WVU III-8 had a VA of 20/50 in the right eye and 20/200 improving with pinhole to 20/50 in the left eye at the age of 77 years, but GC4177: IV-1 suffered severe VA loss during the seventh decade. Not all subjects were available for refraction (Table 2). All three carriers from the WVU pedigree whose visual fields were available showed enlargement of the blind spot. Additionally, one carrier had bilateral nasal and temporal scotomata.

**Figure 2:**
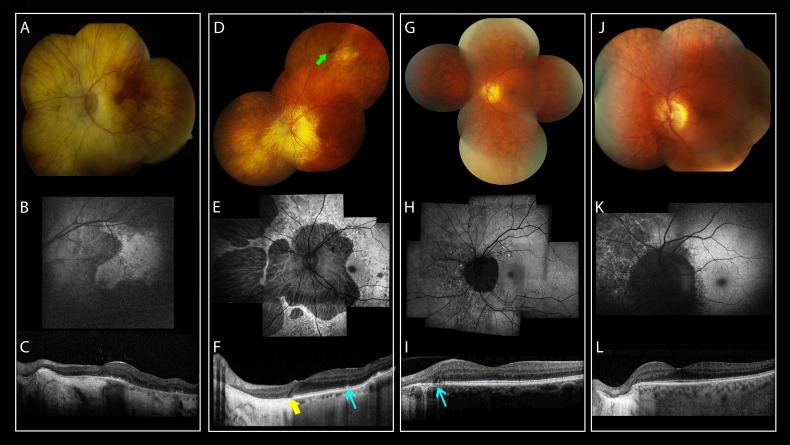
Retinal images for the carriers of the c.836dupG mutation in RGR from the WVU pedigree. Color fundus photographs (A, D, G, J), FAF (B, E, H, K), and OCT scans (C, F, I, L) for individuals from the WVU pedigree harboring the c.824dupG mutation in RGR, each column shows images for one individual. (A–C) Images for patient III-8 showing: (A) diffuse chorioretinal atrophy, with partial preservation of the fovea (note the lack of intraretinal pigment migration); (B) FAF of the posterior pole showing an island of preserved, yet mottled signal; (C) OCT shows loss of the ellipsoid zone (EZ) in the entire scan except for a small area under the fovea, with marked attenuation of the outer nuclear layer (ONL); the choroidal blood vessels are nearly absent. (D–F) images for patient III-6 showing: (D) extensive peripapillary atrophy, and an isolated patch of intraretinal pigmentation (green arrow); (E) FAF shows loss of AF extending in the nasal retina, and a reticular pattern in the posterior pole with foveal sparing; (F) OCT shows an intralaminar bridge (yellow arrow) demarcating the edge of the preserved outer retina; focal excrescence of the RPE band is shown in the region of the reticular changes (cyan arrow). (G–I, J–L) Images for patients IV-5 and IV-6, showing peripapillary atrophy and reticular pigmentation (G, J), peripapillary hypoautofluorescence and reticular changes nasal to the optic disc (H, K), sharp demarcation on OCT between the atrophic and preserved retina (I, L, see cyan arrow in I).

**Figure 3:**
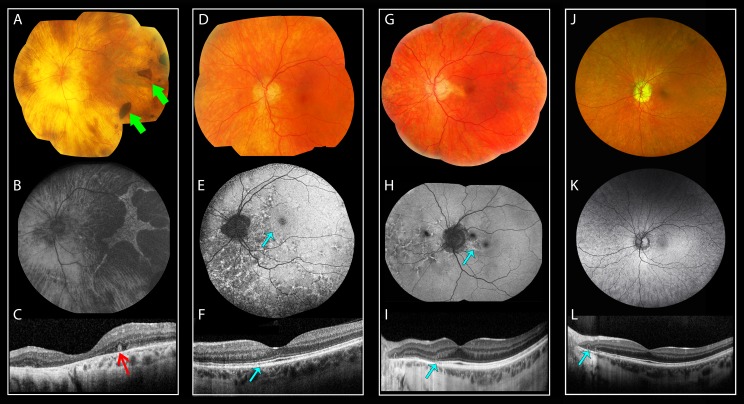
Retinal images for the carriers of the c.836dupG mutation in RGR from the MEH pedigree. Color fundus photographs (A, D, G), FAF (B, E, H), and OCT images (C, F, I) for individuals from the GC4177 family carrying the c.824dupG mutation in RGR. (A–C) Images for the proband IV-1 showing (A) diffuse chorioretinal atrophy, patches of intraretinal pigmentation (green arrows) are noted in the temporal retina; (B) FAF shows diffuse loss of AF except for an island with scalloped edges temporal to the fovea; (C) OCT shows outer retinal tubulation (ORT; red arrow). (D–F) Images for patient V-1. (D) Deep reticular pigmentation in the nasal retina. (E) FAF shows peripapillary atrophy and reticular changes in the nasal retina, note the edge nasal to the fovea corresponding to the subtle irregularity of the ellipsoid zone and RPE bands on OCT ([F], cyan arrow). (G–I) Fundus images for the asymptomatic carrier V-2 showing (G) peripapillary atrophy and reticular pigmentation nasal to the optic disc and temporal to the macula. (H) Outer retinal atrophy extending toward the fovea (cyan arrow), and reticular AF pattern nasal to the disc. (I) OCT showing attenuation of the ONL and EZ nasal to the fovea. Note the ORT temporal to optic nerve head. (J–L) fundus images of the asymptomatic carrier VI-1. (J) peripapillary atrophy and diffuse deep retinal pigmentation anterior to the vascular arcades, better visualized on FAF (K). (L) OCT showing absence of the ONL and EZ temporal to the optic nerve head; note also the ORT (cyan arrow).

Fundus examination of subjects WVU: III-8 and III-6 at ages 77 and 81 years, respectively, and GC4177: IV-1 at age 73 years showed increased visibility of the large choroidal blood vessels, in keeping with loss of the RPE, without significant attenuation of the retinal blood vessels (Figs. 2, 3). Two subjects showed discrete patches of deep retinal hyperpigmentation: WVU: III-6 (Fig. 2D, green arrow) and GC4177: IV-1 (Fig. 3A, green arrows). Subject GC4177: V-1 showed only peripapillary atrophy, but the deep retinal changes became conspicuous on FAF imaging as described below. The asymptomatic carriers (WVU: IV-2, IV-5, IV-6 and GC4177: V-2, VI-1) showed, in addition to peripapillary atrophy, bilateral deep retinal pigmentation anterior to the vascular arcades (Figs. 2G, 2J) and nasal to the optic disc (Fig. 3G). Subject GC4177: V-2 showed peripapillary atrophy extending toward the fovea (Fig. 3G).

FAF imaging showed sharply demarcated areas of hypoautofluorescence in advanced cases. The areas of relatively preserved outer retina had mottling of the AF or reticular hyperautofluorescence (Figs. 2B, 2E, 3B, 3E). The changes were more noticeable on FAF than on color fundus imaging, with FAF showing reticular hyperautofluorescence and patches of reduced AF (Figs. 2E, 2H, 2K, 3H).

OCT showed marked disruption of the inner segment–outer segment junction band (IS/OS) in advanced cases (WVU: III-8 and GC4177: IV-1) with attenuation of the outer nuclear layer (Figs. 2C, 3C). Outer retinal tubulation was present at the junction between the atrophic fovea and a layer resembling the IS/OS (Fig. 3C, red arrow) or temporal to the optic nerve head (Figs. 3I, 3L). Subject WVU: III-6 showed preservation of the central foveal structure on OCT, separated from the nasal, atrophic region by a hyporeflective band reminiscent of an interlaminar bridge (Fig. 2F, yellow arrow). Subject GC4177: V-1 showed mild IS/OS irregularity on OCT (Fig. 3F, cyan arrow). The asymptomatic carriers showed attenuation or loss of the photoreceptor layer in the peripapillary area (Figs. 2I, 2H, 3I). Foveal lamination was normal in all asymptomatic carriers.

**Figure 4:**
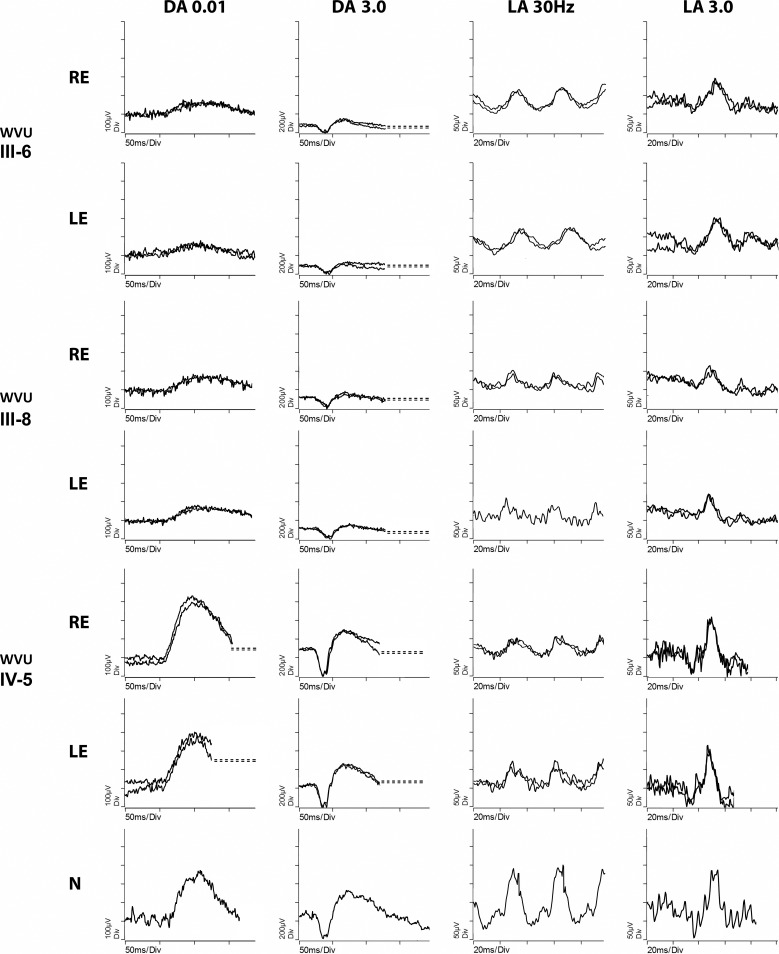
ERG for three carriers of the c.824dupG mutation in RGR: data for WVU: III-6, WVU: III-8, and WVU: IV-5, are presented with responses recorded from a healthy 58-year control in the bottom for comparison. Note that the recording was performed according to the previous ISCEV standard protocols, which have been updated after the recordings were obtained. The WVU age-matched normative data are given in Supplementary Table S2. DA 0.01: dark adapted response to a dim flash stimulus (0.01 cd.s.m^−2^); DA 3.0: dark adapted response to a bright flash stimulus (3.0 cd.s.m^−2^); LA 30Hz: light adapted response to a 30 Hz flicker stimulus with intensity 3.0 cd.s.m^−2^; LA 3.0: light adapted response to a flash stimulus (3.0 cd.s.m^−2^). Note the electrical noise resulting from the mains interference.

**Figure 5:**
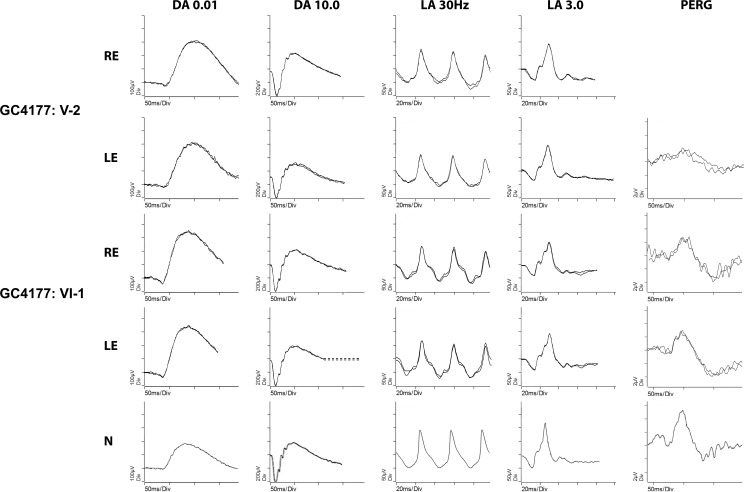
ISCEV-standard ERG for two carriers of the c.824dupG mutation in RGR: GC4177: V-2 and GC4177: VI-1, together with responses recorded from a healthy control in the bottom for comparison. RE, right eye; LE, left eye. DA 0.01: dark adapted response to a dim flash stimulus (0.01 cd.s.m^−2^); DA 10.0: dark adapted response to a bright flash stimulus (10.0 cd.s.m^−2^); LA 30Hz: light adapted response to a 30 Hz flicker stimulus with intensity 3.0 cd.s.m^−2^; LA 3.0: light adapted response to a flash stimulus (3.0 cd.s.m^−2^); PERG: pattern ERG response to a standard 15° field. The full field and PERG for VI-1 are normal. The right eye of V-2 underwent extended dark adaptation before ERG testing to investigate if the RGR mutation affects the visual cycle. The amplitude of the dark-adapted response to DA 10.0 from the right eye is higher than that from the left eye, but this difference is not clinically significant. The scotopic and photopic full field ERGs from both eyes are normal. PERG shows mild reduction of the P50 amplitude in the left eye, with normal peak time.

Electroretinography was available for three subjects from the WVU pedigree—data given in Supplementary Table S1 and waveforms in Figure 4 (one subject also had an EOG)—and two subjects from the GC4177 pedigree (Fig. 5). The asymptomatic carriers WVU: IV-5 (note the normative data in Supplementary Table S2) and GC4177: V-2, VI-1 had normal scotopic and photopic full field ERGs. The PERG was normal in the youngest carrier GC4177:VI-1, but showed mildly reduced P50 amplitude in GC4177: V-2. Subjects WVU: III-6 and WVU: III-8 were symptomatic, and the ERG from WVU: III-6 showed moderately severe reduction of the scotopic amplitudes at the age of 81 years, with delayed and subnormal photopic responses. The ERG recordings from WVU: III-8 (at the age of 59 years with limited outer retinal atrophy) showed moderate reduction of the scotopic bright flash response; and subnormal photopic b-wave amplitude with normal peak time, in addition to mild reduction of the EOG light rise (light peak-dark trough ratio of 1.6) in both eyes. Because of the putative role of RGR in the visual cycle,^13^ ERG was performed in subject GC4177: V-2 after extended monocular dark adaptation overnight. The amplitude of the scotopic mixed bright flash response of the eye that underwent extended dark adaptation was higher than that of the eye that underwent standard dark adaptation for 20 minutes, but this difference was not clinically significant.

## Discussion

We described the clinical features of retinal dystrophy associated with a rare, heterozygous frameshifting mutation in *RGR* in two families from the United States and the United Kingdom with a haplotype that is shared with the first reported family,^8^ and demonstrated the spectrum of retinal changes. These ranged from seemingly innocuous reticular or diffuse deep retinal pigment alteration, normal visual acuity, and normal ERG, to diffuse atrophy of the retina and choroid with severe visual loss. Longitudinal data for the asymptomatic carriers were limited and, therefore, it is unknown if the retinal changes in those subjects are nonprogressive signs of the carrier state or represent an early stage of a slowly progressive retinal dystrophy. The features of *RGR*-retinopathy emphasize the importance of FAF imaging in recognizing mild retinal manifestations and guiding the molecular investigation in individuals with poor vision under dim lighting conditions and peripapillary RPE atrophy. Variable expressivity (variable phenotypic severity) and low penetrance (mutation carriers with no associated phenotype) also have been observed in pedigrees heterozygous for the p.Asp477Gly allele in *RPE65*, another dominant disease affecting the RPE.^13^

In the first report of *RGR* retinopathy, the proband showed bilaterally subnormal VA, macular atrophy, and severely reduced ERGs, but the milder and early manifestations of this retinopathy were not described.^8^ The clinical features of retinal dystrophies with presumed primary RPE pathology differ from those of retinitis pigmentosa by the paucity of intraretinal bone spicule pigment deposition, and relative preservation of the retinal vascular caliber. The phenotype described in patients WVU: III-8 and GC4177: IV-1 resembles that observed in choroideremia (CHM), patients heterozygous for the p.Asp477Gly mutation in *RPE65*, some *PRPH2* mutations, and the p.M216K mutation in *RHO*.^13–16^ CHM, and *RPE65*- and *RGR*-dominant retinopathies affect initially the RPE due to the expression of their respective genes, and, therefore, are likely to have similar clinical features. Early RPE involvement in some cases of *PRPH2*-retinopathy and the p.M216K mutation in *RHO* remain unexplained as both genes are expressed in the photoreceptors.^15,16^ One early symptom common to these disorders is reduced vision in the dark; it is plausible to speculate that the impaired night vision is caused by an abnormal visual cycle in the affected RPE while the cone function is spared due to the Müller cell-mediated visual cycle.^17^

Electrophysiologic testing in one individual with advanced retinopathy showed loss of retinal function without shifting the peak-time, suggesting that the main pathology in some cases is loss of photoreceptor function without dysfunction. The subnormal EOG in this patient can be associated with reduction of the scotopic ERG and is milder than the reported abolished EOG light rise in a patient with a similar retinal phenotype in dominant RPE dystrophy due to the p.Asp477Gly allele in *RPE65*.^13^ *RGR* and *RPE65* are expressed in the RPE and these alleles likely result in cellular toxicity.^5,13,18^ The subnormal macular function, as shown on PERG in one asymptomatic carrier, suggested that this retinopathy can involve the macula.

To date, there are no other known disease-causing mutations in *RGR*. It is informative to examine the population variants in the gnomAD database (available in the public domain at http://gnomad.broadinstitute.org/gene/ENSG00000148604; the Broad Institute). The presence of numerous predicted loss of function mutations suggests that haploinsufficiency is too common to be responsible for retinal disease and supports our hypothesis that the reported allele has a toxic or gain-of-function effect. Moreover, eight instances of one specific final exon frame-shifting mutation in 24028 African alleles also are present (NM_002921.3:c.796_797insCC; p.Ile267Profs*37; rs770085833). No other C-terminal frame-shifting alleles are reported. The c.796_797insCC variant, unlike the c.836dupG variant reported in our study, affects the noncanonical reading-frame and produces a different carboxyl terminal peptide (by an out-of-frame extension through the canonical stop codon, adding seven novel amino-acids at the carboxyl terminal, and replacing 29 amino-acids in the cytoplasmic domain of RGR). In addition to containing distinct amino-acids, it also is shorter than that predicted from the c.836dupG variant. This observation further supported the assertion that the retinopathy reported here is due to toxicity conferred by the specific abnormal carboxyl peptide.

The shared haplotype, in the three pedigrees, suggests that the p.Ile280Asnfs*78 allele is a founder mutation. Recognition of this phenotypic variability could lead to identification of further affected families, and analysis of their respective haplotypes.

Our study reinforced the notion that, although candidate gene testing—when a distinct phenotype is observed—often is helpful, unbiased genetic screening using large NGS panels or whole exome/genome sequencing can reveal unexpected genotype–phenotype associations and expand the list of candidate genes.^19,20^

In conclusion, this report exemplified the success of the unbiased approaches of WGS and NGS in the molecular diagnosis of a highly genetically heterogeneous disorder, such as retinal dystrophy. The clinical features suggested a variable severity of retinopathy consequent upon heterozygosity of a specific allele of the *RGR* gene. The slow progression of this retinopathy and possible primary involvement of the RPE make this disorder tractable to potential therapeutic interventions.

## Supplementary Material

Supplement 1
