# Supplementary material for: Clinical Features of a Retinopathy Associated With a Dominant Allele of the RGR Gene
Source: Invest Ophthalmol Vis Sci. 2018 Oct;59(12):4812–20. doi: 10.1167/iovs.18-25061 (PMC6181194; doi:10.1167/iovs.18-25061)
Supplement: Supplement 1 [file iovs-59-11-12_s01.pdf]

Supplementary material:

Examination	RE		LE		Normal Limits	
**Subject: III-6 (81 yrs)**	**Amplitude (µV)**	**Latency (ms)**	**Amplitude (µV)**	**Latency (ms)**	**Amplitude (µV)**	**Latency (ms)**
<u>Dark-Adapted (DA)</u>						
DA 0.01 (b)	65	92	50	90	32	
DA 3.0 (a)	79	16	66	20	87	26
DA 3.0 (b)	163	49	99	29	283	53
<u>Light-Adapted (LA)</u>						
LA 3.0 (a)	19	15	16	16		
LA 3.0 (b)	65	34	73	34	30	32
LA (30 Hz)	49	33	48	33	18	32
**Subject: III-8 (59 yrs)**						
<u>Dark-Adapted (DA)</u>						
DA 0.01 (b)	86	82	75	84		
DA 3.0 (a)	106	18	97	23		
DA 3.0 (b)	151	47	133	47		
<u>Light-Adapted (LA)</u>						
LA 3.0 (a)	12	20	11	16		
LA 3.0 (b)	43	28	45	28	52	32
LA (30 Hz)	31	29	27	29	33	42
**Subject: IV-5 (49 yrs)**						
<u>Dark-Adapted (DA)</u>						
DA 0.01 (b)	275	83	292	88	103	
DA 3.0 (a)	268	16.5	228	16	146	25
DA 3.0 (b)	475	48	386	47	334	53
<u>Light-Adapted (LA)</u>						
LA 3.0 (a)	54	15	38	16		
LA 3.0 (b)	126	30	147	29	62	33
LA (30 Hz)	41	29	53	29	40	31

Table S2: The amplitudes (μV) and latencies (ms) for the electroretinograms from subjects: WVU: III-6, WVU: III-8 and WVU: IV-5, and normative data from age-matched controls. DA 0.01: dark adapted response to a dim flash stimulus (0.01 cd.s.m^{-2}); DA 3.0: dark adapted response to a bright flash stimulus (3.0 cd.s.m^{-2}); LA 30Hz: light adapted response to a 30 Hz flicker stimulus with intensity 3.0 cd.s.m^{-2}; LA 3.0: light adapted response to a flash stimulus (3.0 cd.s.m^{2}). The a- and b- denote the a-wave, and b-wave respectively.

Supplementary material:

Genetic analysis

Initial candidate gene screening using Sanger sequencing of all exons and exon-intron boundaries was done independently at WVU and MEH for the probands from both families where the DNA from WVU-IV:5 did not reveal any likely disease-causing variants in *PRPH2* and *CHM*. The DNA from GC4177-IV:1 tested negative for mutations in *PRPH2, CHM*, *RPE65* (dominant mutation: p.Asp477Gly), *RHO,* and *C1QTNF5*. The DNA from WVU-IV:5 underwent next-generation sequencing (NGS) as part of the eyeGENE study, the DNA from GC4177-IV:1 underwent whole-genome sequencing (WGS) as part of the NIHRBR-RD study.

<u>Next Generation Sequencing (eyeGENE)</u>

Design of the microdroplet-based PCR primer library

A custom panel of 184 genes, known to be associated with retinal degeneration (http://www.RetNet.org), was used to design primer pairs targeting the coding exons of the 184 genes (available upon request) and their flanking intronic splice regions using a custom primer design pipeline based on the Primer3 algorithm (http://frodo.wi.mit.edu/primer3).

RainDance target enrichment and NGS sequencing

The sheared 5 kb genomic DNA fragments (Covaris M220 instrument: Covaris, Woburn, MA) underwent targeted sequence amplification by PCR. The purified amplicons entered the preparation protocol (NEXTflex PCR-Free DNA Sequencing Kit and NEXTflex PCR-Free barcode 1, Illumina®-Compatible, BIOO Scientific, Austin, Texas), followed by 150 bp pair-end sequencing on a MiSeq2500 instrument (Illumina, San Diego, CA). Samples in each batch were indexed using 12 different index tags (Nextera, Epicentre Biotechnologies).

Variant Detection Analysis

Reference gene sequences were annotated with known single-nucleotide polymorphisms (SNPs) from the NCBI dbSNP database build 130, 1000 Genomes database build 201105 and 201011 and mutations from the Human Gene Mutation Database (HGMD) (http://www.hgmd.cf.ac.uk/) or Leiden Open Variation Database (LOVD) (http://www.lovd.nl/3.0) or reported in the literature. Other variant reference databases used were: HapMap variants database (Ensembl), Clinical Variants in dbSNP database (NCBI), 1000 genomes database (Ensembl), Chromosome bands ideogram (UCSC), dbSNP common variants (UCSC), Genomic Annotations (Ensembl), Cosmic Noncoding Variants & Coding Mutations (cancer.sanger.ac.uk). The sequencing reads were aligned to the reference sequence from NCBI (hg19 Build 37) and analyzed using the CLC Genomics Workbench software ™ (Qiagen) and called using the Probabilistic Variant Detection Tool under robust and stringent sequencing criteria set by the CLC Genomics Workbench with a probability call of 100 to delete any false mutation observations. Identified sequence variants were annotated according to the guidelines published by the Human Genome Variation Society (HGVS).

Whole genome sequencing (NIHRBR-RD)

DNA from 599 unrelated probands with inherited retinal disease, ascertained from the Inherited Eye Disease clinics at MEH, underwent WGS. Peripheral blood mononuclear cell-derived genomic DNA was processed using the Illumina TruSeq DNA PCR-Free Sample Preparation kit (Illumina Inc., San Diego, CA, USA) and sequenced using an Illumina Hiseq 2500, generating minimum coverage of 15X for ~95% of the genome. Reads were aligned to the genome (GRCh37) using Isaac aligner (Illumina Inc, Great Chesterford, UK). Single nucleotide variations (SNVs) and indels were identified using Isaac variant caller. Variant examination was performed only on the SNVs and indels that met the following criteria: passed standard quality filters, predicted to alter the sequence of a protein, and had an allele frequency <0.01 in the 1000 genomes database, the NHLBI GO Exome Sequencing Project (URL: http://evs.gs.washington.edu/EVS/ release 20130513), the UK10K database (http://www.uk10k.org), and the Exome Aggregation

Consortium (ExAC) database (URL: http://exac.broadinstitute.org), and <0.02 in ~6000 internal control genomes. The reads of the whole genome sequences were inspected manually using the Integrated Genome Browser (URL: http://www.broadinstitute.org/igv/home)[1,2].

Initially, likely disease-causing variants in a panel of 192 genes previously associated with inherited retinal disease were interrogated (gene list available on request). Variants were ranked based on previous identification in retinal disease in the literature and/or a predicted impact on protein function, including high pathogenicity scores for missense variants using the predictive algorithms of 'Sorting Intolerant from Tolerant' (SIFT) available at http://sift.jcvi.org and Polymorphism Phenotyping v2 (PolyPhen-2) available at http://genetics.bwh.harvard.edu/pph2[3,4].

Haplotype analysis

WGS data for the proband GC4177: IV-1, heterozygous for the c.836dupG mutation in *RGR*, were interrogated in the region telomeric and centromeric to *RGR*. All SNVs with a reference ID in the refSNP cluster (rs), and a minor allele frequency (MAF) of less than 0.2 in the gnomAD dataset (http://gnomad.broadinstitute.org) were identified. Thirty-two SNVs spanning 8.9 Mb were selected for direct Sanger sequencing in the two affected offspring (V-1 and V-2), and subsequently the unaffected paternal DNA to identify the disease haplotype (reference ID of the SNVs are available upon request).

The disease haplotype SNVs in the GC4177 pedigree were examined by Sanger sequencing in the proband WVU: IV-5 and an affected sibling WVU: IV-6 from the WVU pedigree and three affected individuals and two unaffected individuals from the pedigree reported by Morimura et al[5].

References

1. Thorvaldsdóttir H, Robinson JT, and Mesirov JP. Integrative Genomics Viewer (IGV): high-performance genomics data visualization and exploration. Brief Bioinform. 2013;14(2):178-192.

2. 1000 Genomes Project Consortium, Abecasis GR, Auton A, et al. An integrated map of genetic variation from 1,092 human genomes. Nature. 2012 (7422);491:56-65.

3. Adzhubei IA, Schmidt S, Peshkin L, et al. A method and server for predicting damaging missense mutations. Nat Methods. 2010;7(4):248-249.

4. Kumar P, Henikoff S, and Ng PC. Predicting the effects of coding non-synonymous variants on protein function using the SIFT algorithm. Nat Protoc. 2009;4(7):1073-1081.

5. Morimura H, Saindelle-Ribeaudeau F, Berson EL, and Dryja TP. Mutations in RGR, encoding a light-sensitive opsin homologue, in patients with retinitis pigmentosa. Nat Genet. 1999;23(4):393-4.
